# Prevalence, Risk Factors, and Mortality of New-Onset Atrial Fibrillation in Mechanically Ventilated Critically Ill Patients

**DOI:** 10.3390/jcm13226750

**Published:** 2024-11-09

**Authors:** George E. Zakynthinos, Vasiliki Tsolaki, Andrew Xanthopoulos, Nikitas Karavidas, Vasileios Vazgiourakis, Fotini Bardaka, Grigorios Giamouzis, Ioannis Pantazopoulos, Demosthenes Makris

**Affiliations:** 1 3rd Department of Cardiology, “Sotiria” Chest Diseases Hospital, Medical School, National and Kapodistrian University of Athens, 11527 Athens, Greece; 2 Critical Care Department, University Hospital of Larissa, Faculty of Medicine, University of Thessaly, 41110 Larissa, Greece; vasotsolaki@yahoo.com (V.T.); nikitaskaravidas@gmail.com (N.K.); vasvazg@yahoo.com (V.V.); bardakafotini@yahoo.gr (F.B.); dimomakris@uth.gr (D.M.); 3 Department of Cardiology, University Hospital of Larissa, Faculty of Medicine, University of Thessaly, 41110 Larissa, Greece; andrewvxanth@gmail.com (A.X.); grgiamouzis@gmail.com (G.G.); 4 Department of Respiratory Medicine, Faculty of Medicine, University of Thessaly, 41110 Larissa, Greece; ipantazop@uth.gr

**Keywords:** new-onset atrial fibrillation, critical illness, intensive care unit, mortality, sepsis, septic shock

## Abstract

**Background/**Objectives**:** Critically ill patients admitted to the intensive care unit (ICU) frequently develop new-onset atrial fibrillation (NOAF) due to numerous risk factors. While NOAF has been associated with increased mortality, it remains unclear whether it serves merely as a marker of illness severity or directly contributes to adverse outcome. This study aimed to determine the incidence and risk factors for NOAF in a homogenized population of mechanically ventilated patients at ICU admission, excluding well-established predisposing factors. Additionally, we examined the impact of NOAF on mortality in this context. **Methods:** We prospectively studied consecutive patients over a 3-year period to identify triggers for NOAF. Factors associated with 30-day mortality during the ICU stay were recorded. Demographic data, medical history, laboratory findings, and the severity of illness at admission were compared between patients who developed NOAF and those remaining in sinus rhythm. In NOAF patients, the course of atrial fibrillation (resolution, persistence, or recurrence) was evaluated during the 30-day ICU stay. **Results:** Of the 1330 patients screened, 685 were eligible for analysis, with 110 (16.1%) developing NOAF. Septic episodes occurred more frequently in the NOAF group compared to the no-NOAF group (92.7% vs. 58.1%, *p* < 0.001). Notably, 80% of NOAF patients developed a septic episode concurrently with the atrial fibrillation, often stemming from secondary infections, and 85.3% presented with septic shock. When focusing on patients with at least one septic episode during the 30-day ICU stay, 23.4% of them developed NOAF. Additionally, patients with NOAF were older and had a higher prevalence of hypertension; disease severity at admission was not a triggering factor. Mainly sepsis, but also advanced age, and a history of hypertension remained independent factors associated with its occurrence. Sepsis, primarily, along with advanced age and a history of hypertension, was identified as independent factors associated with the occurrence of NOAF. Mortality was higher in the NOAF group compared to the control group (39 patients (35.5%) vs. 138 patients (24%), *p* = 0.01). NOAF occurrence, sepsis, disease severity at admission, and age were associated with increased ICU mortality; however, NOAF was not found to be an independent predictor of ICU mortality in multivariate analysis. Instead, sepsis, age, and disease severity at admission remained independent predictors of 30-day mortality. Sinus rhythm was restored in 60.9% of NOAF patients within 48 h, with the improvement or stabilization of sepsis being crucial for rhythm restoration. **Conclusions:** NOAF is a common complication in intubated ICU patients and is independently associated with sepsis, advanced age, and hypertension. While NOAF is linked to increased ICU mortality, it is more likely a marker of disease severity than a direct cause of death. Sepsis improvement appears critical for restoring and maintaining sinus rhythm.

## 1. Introduction

New-onset atrial fibrillation (NOAF) is the most common arrhythmia observed in critically ill patients, with reported incidence rates ranging from 4% to 46% [1,2,3,4,5,6,7,8]. NOAF can result in rapid ventricular rates, which reduce cardiac output and lead to hemodynamic instability. This poses a significant risk to already vulnerable patients in the ICU, potentially worsening their condition [9,10]. Therefore, identifying patients at high risk for NOAF is crucial in the critical-care setting.

Although numerous studies have documented the occurrence of NOAF in critically ill patients [2,3,11,12,13], data on the specific risk factors contributing to its development remain limited, with significant variation across different critical-care populations. During ICU admission, NOAF can be triggered by several factors, including vasopressor use, electrolyte imbalances, hemodynamic instability, and, most notably, severe sepsis, which often encompasses all of these conditions [9]. Sepsis is known to cause both structural and electrical abnormalities in the heart, further promoting the development of NOAF [9]. One study reported that 35% of NOAF cases occurring during hospitalization were related to acute infections [14], while an older study found NOAF in 46% of patients with septic shock in a general ICU [3]. This growing body of evidence supports the idea that sepsis is a significant contributor to the onset of NOAF in critically ill patients.

Despite the association between NOAF during critical illness and poor outcomes, including increased mortality [15,16,17,18,19], it remains unclear whether this increased mortality is a direct consequence of AF or merely a marker of the severity of the underlying disease [8,13,16,20,21,22,23,24,25,26]. The higher susceptibility to NOAF is likely driven by the severity of the critical illness, combined with the presence of multiple predisposing and precipitating factors. Additionally, since sepsis is a prevalent diagnosis in all ICUs and significantly influences clinical outcomes, it is crucial to consider this factor in studies assessing the prognostic effects of NOAF.

Therefore, in this prospective observational study, we aimed to evaluate a cohort of critically ill patients who were mechanically ventilated at least upon admission to (1) determine the incidence of NOAF and investigate possible risk factors for its occurrence; (2) examine the course of NOAF during ICU stay; and (3) assess its impact on ICU mortality in a general ICU setting.

## 2. Methods

### 2.1. Study Population

This prospective study included consecutive patients aged 18 years or older admitted to the ICU at the University Hospital of Larissa, Greece, between 1 January 2020 and 31 December 2022. The study was approved by the local ethics committee (20/16th/26 September 2019) with a waiver of informed consent. All patients were mechanically ventilated, with intubation performed either in the emergency department, the general ward, or within the first 24 h of ICU admission; continuous mechanical ventilation throughout the ICU stay was not required for inclusion. The study period extended from the first day of ICU admission until day 30, whether the patient remained in the ICU, was discharged, or died. Thus, 30-day mortality was defined as death from any cause within 30 days of ICU admission. Patients discharged alive from the ICU before 30 days were considered survivors.

### 2.2. Exclusion Criteria

Patients meeting multiple exclusion criteria were assigned to a single category, determined by the attending physician based on the perceived severity of their condition.

Patients were excluded from the study if they had (1) permanent atrial fibrillation (AF) with or without heart failure; (2) history of paroxysmal AF; (3) known history of cardiac disease well-known to predispose to AF, including (a) history of heart failure from any cause or previous echocardiographic findings indicating Left Ventricular Ejection Fraction below 45%, (b) known Right Ventricular Dysfunction, (c) cardiomyopathy of any type, (d) severe valvulopathy: aortic and mitral stenosis or regurgitation, (e) presence of a pacemaker, (f) congenital heart disease; (4) history of severe lung disease; (5) NOAF present at ICU admission; (6) death within the first 24 h of ICU admission; (7) ICU admission due to COVID-19; (8) discharge from the ICU within 48 h (primarily surgical cases); (9) recent myocardial infarction (MI) and/or coronary artery bypass graft surgery (CABG) or coronary artery disease (CAD) unstable or symptomatic (which should have been treated by intervention, but was not). However, patients with a history of old MI, CABG, or stable-asymptomatic or treated by intervention CAD without heart failure or other cardiac disease were included; (10) non-intubated patients.

### 2.3. Study Groups

Eligible patients were divided into two groups: the NOAF group, which included patients who developed new-onset atrial fibrillation during their ICU stay, and the control group, consisting of all other patients who did not experience AF. Patients were included in the NOAF group if they had at least one AF episode lasting more than 5 min, experienced multiple AF episodes within a 24 h period, or required electrical cardioversion due to hemodynamic instability.

### 2.4. Data Collection

Baseline characteristics and disease severity (APACHE II and SOFA scores) were recorded upon ICU admission. Demographic information and data on medical history prior to hospital admission were collected from patients’ medical records and/or their next of kin. Daily laboratory findings, including complete blood count, biochemistry, electrolytes, and C-reactive protein, were documented. Blood, urine, and endotracheal aspirate cultures were collected upon admission and whenever clinically indicated, including on the day of AF onset. Heart rhythm was assessed continuously from the patients’ monitor (General Electric, Carescape B850, GE Healthcare, Chicago, IL, USA); ECG tracings (12 lead) could be reviewed for the preceding 72 h (GE monitor’s software, CSCS—CARESCAPE Software Control System version 2.4), while 12-lead ECG was performed daily. All patients received prophylactic anticoagulation unless contraindicated (e.g., due to coagulopathy, thrombocytopenia, or active bleeding).

### 2.5. Definitions

Sepsis, septic shock, and infection types were defined according to the Sepsis-3 guidelines [27,28]. Sepsis at admission was defined as the presence of sepsis or septic shock, as well as “suspected infection”, characterized by the administration of parenteral antibiotics and sampling of body fluid cultures prior to ICU admission [29] and in keeping with the Sepsis-3 definitions [27]. Suspected infection at admission was confirmed after ICU admission through necessary tests conducted, as well as those that were taken prior to admission and received during the patient’s stay in the ICU. This confirmation was made through laboratory tests, cultures, or other diagnostic methods. Secondary infections included all bloodstream infections (BSIs), ventilator-associated pneumonia (VAP), and urinary tract infections that developed after 48 h of hospital or ICU admission. Confirmation of these infections occurred later in the ICU, once culture results and other diagnostic tests became available. Secondary infections were not the reason for ICU admission; rather, they resulted from complications related to the primary illness.

### 2.6. Atrial Fibrillation Management

Once NOAF was detected, the decision to pursue electrical cardioversion, pharmacological treatment, or a “wait-and-see” approach was made by the attending clinician. However, according to the standard in our ICU practice, NOAF episodes are typically treated with amiodarone (600–750 mg daily), following a loading dose of 150–300 mg. Beta-blockers are often administered initially for rate control. Direct electrical cardioversion is reserved for patients with hemodynamic instability, defined as a significant increase in vasopressor requirements after the onset of AF. Clinicians are encouraged to delay electrical cardioversion until the amiodarone loading dose has been administered.

## 3. Statistical Analysis

Continuous variables were expressed as mean ± standard deviation (SD) when normally distributed, and as median (interquartile range) when not. Categorical variables were presented as percentages. Normality was assessed using the Kolmogorov–Smirnov test. Comparisons between continuous variables were performed using the *t*-test for normally distributed data, or the Mann–Whitney U test for non-normally distributed data. Categorical variables were compared using the chi-square test or Fisher’s exact test, as appropriate. All statistical tests were two-tailed, with a *p*-value of <0.05 considered statistically significant.

Variables identified as significant predictors of NOAF were further analyzed using multivariate logistic regression, with NOAF as the dependent variable. Similarly, variables found to be significant predictors of death in univariate analysis were included in a multivariate logistic regression model, with death as the dependent variable.

All statistical analyses were performed using SPSS version 26.0 (IBM, Armonk, NY, USA).

## 4. Results

**Figure 1:**
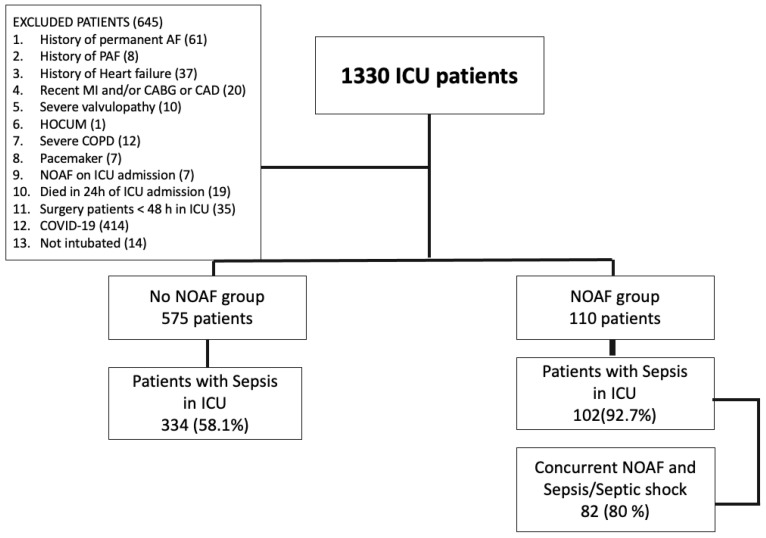
Study Flowchart, AF, Aatrial fibrillation; NOAF, new-onset atrial fibrillation; PAF, paroxysmal atrial fibrillation; MI, myocardial infarction; CABG, coronary artery bypass grafting; CAD, coronary artery disease untreated (which should have been treated by intervention, but was not); HOCM, hypertrophic obstructive cardiomyopathy; COPD, chronic obstructive pulmonary disease; ICU, intensive care unit.

Among 1330 critically ill patients admitted to the ICU over a three-year period, 685 patients met the eligibility criteria for the study (Figure 1). New-onset atrial fibrillation developed in 110 patients (16.1%) during their ICU stay, comprising the NOAF group, while the remaining 575 patients (83.9%) constituted the control group.

The characteristics of the patients are presented in Table 1. Patients who developed NOAF were older and had a higher prevalence of hypertension. Disease severity tended to be higher in the NOAF group as suggested by the APACHE II score (*p* = 0.06); the SOFA score, which does not take in account age as a factor, did not significantly differ between the two groups. In addition, there was no difference in the presence of significant coronary artery disease, as indicated by a history of previous old MI without heart failure, no recent CABG, or stable–asymptomatic CAD.

### 4.1. Septic Episodes and NOAF

At least one septic episode, including cases of sepsis or septic shock at admission, occurred in 334 patients (58.1%) in the control group and 102 patients (92.7%) in the NOAF group (*p* < 0.001) (Figure 1). When considering only patients who experienced at least one septic episode during their 30-day ICU stay, NOAF occurred in 23.4% of cases.

In the NOAF group, 82 patients (80%) experienced a septic episode coinciding with the onset of NOAF, as determined by clinical, laboratory, and/or microbiological findings (Figure 1). Of these, 70 patients (85.3%) presented with septic shock requiring increased doses of vasopressors. Primary bacteremia, associated with the septic episode, was identified in 36 patients (43.9%)—including three cases of fungemia—while 40 patients (48.8%) developed VAP, with or without bacteremia. Notably, antibiotics were initiated or modified in the majority of NOAF patients either on the day of NOAF onset or during the two preceding days, based on suspected septic infections as determined by the treating physicians, regardless of microbiological findings. NOAF typically developed rather late in the ICU stay, with an average onset at 8.6 ± 4.1 days. Only 10 out of 110 patients (9.1%) developed NOAF within the first 48 h, with 7 of these cases classified as hospital-acquired. Thus, most of the septic episodes were categorized as secondary infections. Difficult-to-treat (DTR) multidrug-resistant Gram-negative organisms, including *Klebsiella pneumoniae*, *Acinetobacter baumannii*, and *Pseudomonas aeruginosa*, were the most frequently isolated pathogens from microbiological cultures.

Interestingly, 183 patients (27.4%) presented with sepsis upon ICU admission (Table 1). In this subpopulation of septic patients at admission, the severity of illness, as measured by APACHE II and SOFA scores, did not significantly differ between those who developed NOAF and those who did not. The same was true for the rest patients (without sepsis upon ICU admission). Furthermore, among the patients with sepsis at admission, the incidence of NOAF during the ICU stay was 18.03% (33 out of 183 patients), while the respective incidence in patients without sepsis at admission was 15.33% (77 out of 502 patients) (*p* = 0.46).

### 4.2. Predictors of NOAF

Multivariate logistic regression was performed to identify independent predictors of NOAF. Variables from demographics and baseline characteristics upon ICU admission from Table 1 with *p* < 0.05, along with the presence of at least one septic episode in ICU stay (Figure 1), were included in the multivariate model. Sepsis was the strongest independent predictor of NOAF (Odds Ratio (OR) 11.53, 95% CI 6.53–20.36, *p* < 0.001), while advanced age (OR 1.06, 95% CI 1.03–1.09, *p* < 0.01) and a history of hypertension (OR 1.51, 95% CI 1.03–2.23, *p* = 0.034) were also independently associated with the development of NOAF.

### 4.3. Mortality

ICU mortality was observed in 177 patients (25.8%) in the three-year period studied. Mortality was significantly higher in the NOAF group compared to the control group (39 patients (35.5%) vs. 138 patients (24%), *p* = 0.01). Factors associated with increased ICU mortality included the APACHE II score (OR 1.10, 95% CI 1.07–1.13, *p* < 0.001), the SOFA score (OR 1.08, 95% CI 1.04–1.12, *p* < 0.001), the presence of sepsis (OR 3.32, 95% CI 2.21–5.00, *p* < 0.001), and age (OR 1.06, 95% CI 1.04–1.08, *p* < 0.001). NOAF was also significantly associated with increased mortality (OR 1.72, 95% CI 1.13–2.63, *p* = 0.01) (Table 2). A history of hypertension, old MI/old CABG/stable CAD, gender, potassium concentration, and noradrenaline dose did not impact mortality. However, it is important to note that potassium concentration and noradrenaline dose were assessed at the time of ICU admission.

Multivariate analysis identified sepsis, age, and disease severity at ICU admission (measured by both APACHE II and SOFA scores), as independent predictors of mortality (Table 2). Only variables that were significantly associated with mortality in the univariate analysis were included in the multivariable logistic regression model for ICU mortality. Notably, NOAF was not found to be an independent predictor of ICU mortality.

### 4.4. Outcome–Restoration of Sinus Rhythm

Sinus rhythm (SR) was restored in 60.9% of NOAF patients within 48 h and in 49.1% of patients within the first 24 h. Nearly all NOAF patients (except for nine) were treated with amiodarone infusion. In six patients with severe hemodynamic instability, NOAF was treated with electrical cardioversion. Although atrial fibrillation recurred shortly in five of these patients, they maintained relative hemodynamic stability under amiodarone infusion, and only one required repeat electrical cardioversion. In most cases, SR was re-established once sepsis began to be controlled, even in patients where SR was delayed beyond 48 h.

In sixteen patients (14.5%) where SR was not restored, signs of sepsis (due to DTR secondary infection) persisted until death, with vasopressors and lactate levels remaining uncontrolled. Nine of these patients died within 48 h of NOAF onset due to refractory septic shock.

Ten patients experienced AF recurrence 3–12 days after initial restoration of SR, typically coinciding with a new septic episode. Sinus rhythm was restored in eight of these patients once sepsis resolved.

No embolic complications were observed in any patients, including those who maintained SR after NOAF, experienced NOAF relapse, or had persistent atrial fibrillation. However, detailed imaging assessments (e.g., CT scans of the brain, abdomen, etc.) were not performed in all patients.

## 5. Discussion

In this comprehensive observational study involving patients who required mechanical ventilation for at least an initial period in the ICU, we determined (a) the prevalence of NOAF and its association with existing risk factors in patients admitted to our tertiary general ICU, and (b) the 30-day ICU mortality rate for patients with or without NOAF.

We recorded a NOAF incidence of 16.1% within our ICU population. Factors independently associated with NOAF included advanced age, a history of hypertension, and, most significantly, the presence of sepsis during the ICU stay. NOAF incidence was increased to 23.4% when only patients with at least one septic episode were considered. Notably, the severity of illness at admission was not found to be a significant trigger for NOAF. NOAF often occurred concurrently with a septic episode, typically septic shock, later in the ICU course, suggesting that a secondary infection led to severe sepsis. The resolution of sepsis was crucial in restoring and maintaining sinus rhythm.

Patients with NOAF appeared to have a significantly worse prognosis compared to those without it. However, when ICU mortality risk factors were included in the multivariate analysis, NOAF was not identified as a significant predictor of mortality. Instead, sepsis—most often in the form of septic shock—emerged as the primary independent predictor of ICU death, with disease severity at admission and patient age also independently contributing to mortality risk (Table 2).

### 5.1. Prevalence and Risk Factors for NOAF in the ICU

NOAF was a relatively common manifestation in our ICU, aligning with findings from other cohorts with mixed ICU populations [2,5,6,7,8]. However, the incidence of NOAF in critically ill patients varies significantly across studies, likely due to differences in sample size, patient populations, illness severity, and study design. As a result, the reported prevalence of NOAF ranges from 4.48% to 12.5% in some studies conducted in medical ICUs [1,30,31,32] and from 4.5% to 11.5% in a few studies from mixed ICU settings [7,19,26,33,34,35]. Many studies, however, do not clearly indicate whether septic patients were included, or when they do, the proportion of patients with sepsis or septic shock is often low. For instance, in Fernando et al.’s study, 10.3% of critically ill patients developed NOAF in the ICU, but only 12.9% of the population had sepsis, and just 8.6% experienced septic shock [24].

Notably, the prevalence of NOAF is consistently higher—regardless of the ICU type—when studies specifically include septic patients [2,6,8]. As the proportion of septic patients increases, the incidence of NOAF rises correspondingly, with sepsis being the primary triggering factor [9,36,37]. Therefore, when focusing on sepsis alone, especially septic shock, NOAF rates climb dramatically. In septic patients, NOAF incidence ranges from 23% to 40% [33,38,39] and is even higher among patients with septic shock [3,40]. Meierhenrich’s study reported a 46% NOAF incidence in septic shock patients [3], and Guenancia et al. found a similar prevalence of 44% [40].

In line with these findings, sepsis—usually septic shock—was the strongest independent predictor of NOAF in our study. Eighty percent of patients who developed NOAF did so during a septic episode, and 85.3% of these cases occurred in patients with septic shock. Interestingly, sepsis present at admission was not associated with an increased NOAF incidence. NOAF tended to develop later during the ICU stay, often coinciding with secondary infections caused by drug-resistant bacteria, which are difficult to treat (DTR) and lead to a prolonged period of instability until the infection is controlled—if it can be managed at all. This late-onset NOAF, associated with secondary sepsis from resistant microbes, was also observed in patients with ARDS due to COVID-19 in our recent study [41]. Furthermore, a small subset of patients (10 individuals) experienced a recurrence of NOAF after initially being converted to sinus rhythm, with the relapse coinciding with a new septic episode caused by a secondary infection.

NOAF has been reported to occur more frequently in patients receiving vasopressor agents, in those with electrolyte imbalances, and in patients with greater disease severity [9,18,25,41]. It has also been linked to the presence of pulmonary artery catheters, mechanical ventilation, increased fluid overload, and dehydration [42,43]; epinephrine and norepinephrine, in particular, can lead to increased atrial ectopic discharges, triggering new-onset AF [9].

However, in our study, all patients with septic shock were receiving vasopressor agents, and electrolyte disturbances—mainly hypokalemia—were not significantly different between the two groups at admission. Since NOAF presented later during the ICU stay, potassium levels are corrected, as K^+^ concentration is measured at least twice per day in these ICU patients, allowing for the prompt correction of any even minor derangements. Yet, dehydration is common in septic patients, despite an often-elevated fluid balance. Additionally, all of our patients were on mechanical ventilation for a considerable portion of their ICU stay, while pulmonary artery catheters are rarely used anymore. Therefore, many of the risk factors identified in previous studies for NOAF are common among ICU patients, especially those with sepsis, where these factors often overlap.

Infection and inflammation can lead to accelerated cardiac remodeling [44]. Animal studies of pneumonia have shown that bacteria can invade the myocardium, creating an arrhythmogenic substrate [45]. Sepsis-induced myocardial dysfunction, or sepsis cardiomyopathy, is common [46], with or without reduced systolic function [47]. We suggest that sepsis, combined with endogenous catecholamine surges and the administration of exogenous catecholamines–vasopressors (as in septic shock), can ultimately trigger AF in a heart already compromised by sepsis.

In our study, disease severity at admission was not a significant factor for NOAF, likely because NOAF appeared later during the ICU stay, predominantly in patients with septic shock, where severity is inherently elevated [27]. To our knowledge, few studies have reported on the timing of NOAF after admission. When the timing of NOAF was specified, and severity at admission was identified as a contributing factor, NOAF typically occurred very soon after admission. For instance, in the study by Fernando et al. [24], the median time from hospitalization to the development of NOAF was just 1 day (IQR 1–3). In such cases, it is logical that initial disease severity (including sepsis) would influence the onset of NOAF.

In addition to sepsis, advanced age and a history of hypertension were independent risk factors for NOAF in our study. Older patients are more likely to have comorbidities, including hypertension, which can predispose them to dysrhythmias during their ICU stay [34]. Previous studies have also reported hypertension, either alone or in association with heart disease, as a significant risk factor for NOAF [1,48]; in our study, we excluded patients with heart failure or severe valvular disease, as these are well-established risk factors for AF. Nonetheless, the relationship between hypertension and NOAF remains controversial [49].

### 5.2. ICU Mortality

In our study, the mortality rate was higher in the NOAF group compared to the control group (35.5% vs. 24%), consistent with findings from previous studies. Most of these studies have shown that critically ill patients with NOAF tend to have higher mortality rates, although many did not account for confounding factors [1,5,30].

The clinical significance of NOAF associated with critical illness remains uncertain [50]. It is possible that NOAF acts as a marker of disease severity rather than a direct cause of poor prognosis, identifying patients at higher risk of death without necessarily contributing to it. Some cohort studies have demonstrated an independent association between NOAF and mortality after adjusting for disease severity [6,18,19,20,23,31], while others have not [8,13,16,26]. The conflicting results may be attributed to differences in study design, sample sizes, patient populations, and methods used to assess NOAF (such as prospective vs. retrospective studies or variations in how NOAF is measured).

In our study, which included only intubated patients at admission, sepsis and septic shock were independently associated with mortality. The severity at admission and advanced age were the other independent predictors of ICU mortality (Table 2). Therefore, NOAF appeared to be a consequence of the severity of the disease rather than a factor influencing mortality.

A recent study involving 15,000 critically ill patients found no direct association between NOAF and increased hospital mortality among the entire cohort. However, an interaction was noted between NOAF and sepsis, and the presence of both was associated with higher odds of hospital mortality than either alone [24]. It is important to note that septic patients constituted only a small proportion of the overall population in this study, making it difficult to determine whether NOAF is independently associated with mortality; more notably, it is unclear whether patients with both NOAF and sepsis were more severely ill than those with either condition alone. Consistent with our findings, among patients who developed NOAF, predictors of hospital mortality included advanced age, the severity of illness, a history of CHF (these patients were not included in our study), and persistent AF following treatment (without specifying the reason why the AF was not restored) [24].

### 5.3. NOAF Outcome

Rhythm control has been shown to be more beneficial than rate control in ICU patients [37]. Therefore, another point worth discussing is the restoration of SR following NOAF. In our study, most patients returned to SR through pharmacologic cardioversion using amiodarone; however, AF could not be reversed within 48 h in patients with non-resolving sepsis, where vasopressor requirements were either unchanged or often increased. Additionally, some patients died with persistent AF due to the inability to control the infection. In contrast, among patients where SR was delayed beyond 48 h, restoration generally occurred once sepsis was adequately controlled.

To our knowledge, this relationship has not been thoroughly investigated in the existing literature. Liu et al. [14] conducted a study involving 503 septic patients, categorizing them into those with NOAF (240 patients) and those without (263 patients). Among the NOAF group, 165 patients successfully restored sinus rhythm (NOAF to SR), while 75 experienced persistent or recurrent atrial fibrillation (NOAF to AF) beyond 7 days. Notably, patients in the NOAF to SR group had significantly higher SOFA and APACHE II scores compared to those without NOAF, with severity being even greater in the NOAF to AF group. While it is reasonable to infer that patients who did not restore SR after 7 days were more severely septic at the time of ICU admission or during their stay, the available data do not provide information on the effectiveness of infection treatment or the progression of sepsis in relation to AF restoration in either NOAF group. Furthermore, there are no data on whether AF recurrence correlated with new septic episodes.

Moreover, in the study of Liu et al., the researchers reported a 61.3% in-hospital mortality rate among septic patients whose NOAF did not convert to SR, compared to 26.1% in those whose NOAF restored to SR and 17.5% in septic patients who remained in continuous SR [14]. It is possible, yet not stated, that the inability to manage sepsis was the underlying cause of the failure to restore SR, ultimately leading to increased mortality. This aligns with the previous paragraph, suggesting that NOAF may be a marker of severe or uncontrolled sepsis rather than an independent predictor of mortality. In Fernando et al.’ study among NOAF patients, 22.4% had sustained AF, defined as lasting more than 24 h [24]. However, there remains a lack of information on the duration of NOAF and whether it was related to severe sepsis or a failure of sepsis to respond to treatment.

In our research, only six patients (5.5% of those with NOAF) experiencing severe hemodynamic instability underwent electrical cardioversion, aligning with Liu et al.’s findings, which reported cardioversion in eight septic patients (3.3%) with NOAF [14].

In summary, advanced age, a history of hypertension, and the presence of sepsis during the ICU stay were independently associated with the occurrence of NOAF. In many cases, AF could not be reversed within 48 h in patients with non-resolving sepsis, where vasopressor requirements remained unchanged or often increased. Ongoing sepsis, combined with endogenous catecholamine surges and the administration of exogenous catecholamines-vasopressors, maintained the AF.

Indeed, a score that could predict the likelihood of cardioversion with specific drugs, certain sepsis indicators, and potentially other factors could help physicians tailor AF management. Recently, Mariani et al. developed and validated a score to predict spontaneous conversion (SCV) to sinus rhythm in patients presenting to the emergency department with hemodynamically stable, symptomatic atrial fibrillation. The proposed score enabled the researchers to predict SCV probability during a 6 h “wait-and-watch” approach with good accuracy. However, critically ill patients were excluded, and no information is provided about sepsis in the included patients. Moreover, a first episode of AF, i.e., NOAF, was noted in only half of the patients [51]. Additionally, since AF is the most frequent arrhythmia leading to emergency department visits, the researchers included a very large number of patients, which allowed for a more comprehensive evaluation of the proposed score. It is possible that a similar score for ICU patients may be developed in the future. Until then, we believe that the effective management of sepsis, particularly septic shock, is crucial for the rapid restoration of sinus rhythm.

### 5.4. Study Limitations and Strengths

Our study has some limitations. First, it was conducted at a single center serving an urban population, which may limit the generalizability of our findings. Future studies should aim to validate these results across multiple ICUs to ensure broader applicability.

Despite this limitation, our study includes a sufficiently large sample size, consisting of prospectively enrolled, severely ill patients over a three-year period. This is particularly relevant for Southern Europe and other regions with a high incidence of DTR nosocomial infections, such as in our region. Secondary infections with multidrug-resistant or even extensively drug-resistant (XDR) and pan-drug-resistant (PDR) Gram-negative microorganisms—all challenging to treat—could significantly contribute to the development of NOAF, especially given the difficulties in controlling sepsis and ultimately restoring sinus rhythm.

Another notable strength of our study is that, to the best of our knowledge, it is the first comprehensive scholarly effort to determine the incidence of NOAF specifically in intubated ICU patients. Furthermore, the patient population can be considered homogeneous, as we prospectively enrolled consecutive intubated patients upon admission and excluded those with known significant pre-existing cardiovascular disease that could trigger AF.

One additional limitation is that echocardiography was not performed in all patients, particularly at the time of NOAF occurrence. As a result, we could not include echocardiographic findings such as ventricular systolic and diastolic function, atrial dimensions, cardiac cavity pressures (assessed indirectly through echocardiography), or the diagnosis of septic cardiomyopathy in some patients, potentially influencing the onset or persistence of NOAF.

## 6. Conclusions

This study found that mainly sepsis, along with advanced age and a history of hypertension, was independently associated with the development of NOAF. NOAF typically emerged later during the ICU stay, often coinciding with secondary sepsis and frequently associated with septic shock. The resolution of sepsis was crucial for maintaining sinus rhythm.

Although NOAF was linked to an increased risk of ICU mortality, it was the severity of illness at admission, the presence of sepsis, and advanced age—not NOAF itself—that were independently associated with mortality. This suggests that while NOAF may serve as a marker of disease severity, it does not independently contribute to mortality. Moreover, in our study, secondary infections caused by XDR and PDR microorganisms—both of which pose significant challenges in controlling sepsis—led to an increased number of patient deaths. In fact, the development of NOAF was an indication of severe sepsis, akin to a clinical sign of serious illness. This may explain why NOAF did not remain an independent predictor in the multivariate analysis despite its strong correlation with mortality in the univariate analysis.

Given that other studies have identified NOAF as an independent risk factor for death in ICU patients, the question of whether this relationship is truly causal remains controversial.

Therefore, further prospective randomized trials are needed to determine whether NOAF is an independent predictor of mortality or simply a marker of severe illness in ICU patients. These studies should focus on specific subgroups of critically ill patients, such as those who are intubated and mechanically ventilated and/or septic, potentially differentiating between primary and secondary infections, to yield more reliable conclusions.

## Figures and Tables

**Table 1:** Demographics and baseline characteristics in patients without AF (controls) and NOAF patients upon ICU admission.

	Control (*n* = 575)	NOAF (*n* = 110)	*p*
Demographics			
Age (years ± SD)	61.1 ± 7.5	65.3 ± 7.1	<0.001
Males N (%)	362 (63%)	74 (67.3%)	0.36
BMI, kgr/m^2^	27.4 ± 7.2	27.9 ± 6.4	0.76
Hypertension N (%)	223 (38.8%)	53 (48.2%)	0.02
History of MI/CABG/CAD (%)	76 (13.2)	15 (13.6)	0.91
Clinical Data			
APACHE II (±SD)	16.6 ± 5.7	17.7 ± 5.4	0.06
SOFA (± SD)	8.1 ± 5.0	8.4 ± 5.8	0.56
K^+^ (mEq/lit ± SD)	3.7 ± 0.8	3.8 ± 0.9	0.23
Noradrenaline (μg/kg/min ± SD)	0.12 ± 0.9	0.14 ± 0.11	0.6
CRP (mg/dL) (<0.5)	2.2 ± 0.8	2.6 ± 1.6	0.06
Sepsis at Admission			
Patients N (%)	150 (26.1%)	33 (30%)	0.4
APACHE II (±SD)	17.3 ± 7.1	18.1 ± 6.0	0.54
SOFA (±SD)	8.2 ± 4.7	8.5 ± 6.1	0.75
CRP (mg/dL), (<0.5)	3.6 ± 1.3	3.9 ± 1.0	0.41
Without Sepsis at Admission			
Patients N (%)	425 (73.9%)	77 (70%)	0.39
APACHE II (±SD)	16.35 ± 5.13	17.53 ± 5.15	0.064
SOFA (±SD)	8.06 ± 5.11	8.36 ± 5.71	0.64

APACHE II, Acute Physiology and Chronic Health Evaluation II; NOAF, new-onset atrial fibrillation; SOFA, Sequential Organ Failure Assessment; MI/CABG/CAD, myocardial infarction/coronary artery bypass graft surgery/coronary artery disease (stable: after angioplasty or not requiring percutaneous coronary intervention (PCI)); BMI, body mass index; K^+^, potassium ion; CRP, C-reactive protein; ICU, intensive care unit. *p* < 0.05 for comparisons between the control and NOAF group upon ICU admission.

**Table 2:** ICU mortality. Univariate and multivariate logistic regression analyses including common predictors.

Univariate Logistic Regression Analysis for ICU Mortality			
Variable	Odds Ratio (95% CI)		*p*
NOAF	1.72 (1.13–2.63)		0.01
AGE	1.06 (1.04–1.08)		<0.001
APACHE II	1.10 (1.07–1.13)		<0.001
SOFA score	1.08 (1.04–1.12)		<0.001
K^+^	0.96 (0.73–1.27)		0.77
Noradrenaline	1.03 (0.55–2.77)		0.62
History of hypertension	1.03 (0.69–1.53)		0.88
History of MI/CABG/CAD	1.02 (0.58–1.79)		0.94
Sepsis	3.32 (2.21–5.00)		<0.001
Males	1.10 (0.77–1.56)		0.61
**Multivariable Logistic Regression Analysis for ICU Mortality**			
**Variable**	**Odds Ratio (95% CI)**	**b Coefficient** **(95% CI)**	***p***
NOAF	1.32 (0.80–2.18)	0.28 (−0.22–0.78)	0.27
AGE	1.04 (1.02–1.07)	0.043 (0.018–0.067)	0.001
APACHE II	1.08 (1.05–1.11)	0.076 (0.053–0.099)	<0.001
SOFA score	1.04 (1.00–1.08)	0.039 (0.000–0.078)	0.047
Sepsis	2.59 (1.65–4.06)	0.95 (0.50–1.40)	<0.001

APACHE II, Acute Physiology and Chronic Health Evaluation II; NOAF, new-onset atrial fibrillation; SOFA, Sequential Organ Failure Assessment; MI/CABG/CAD, myocardial infarction/coronary artery bypass graft surgery, coronary artery disease (stable); K^+^, potassium ion, CI, confidence interval; ICU, intensive care unit.

## Data Availability

The datasets used and analyzed during the current study are available from the corresponding author on request.
